# Electromagnetic wave absorption and compressive behavior of a three-dimensional metamaterial absorber based on 3D printed honeycomb

**DOI:** 10.1038/s41598-018-23286-6

**Published:** 2018-03-19

**Authors:** Wei Jiang, Leilei Yan, Hua Ma, Ya Fan, Jiafu Wang, Mingde Feng, Shaobo Qu

**Affiliations:** 1 Science College, Air Force Engineering University, Xi’an, 710051 China; 2 State Key Laboratory for Strength and Vibration of Mechanical Structures, Xi’an Jiaotong University, Xi’an, 710049 China

## Abstract

Lightweight structures with multi-functions such as electromagnetic wave absorption and excellent mechanical properties are required in spacecraft. A three-dimensional metamaterial absorber consisting of honeycomb and resistive films was proposed and fabricated through 3D printing and silk-screen printing technology. According to simulation and experiment results, the present three-dimensional metamaterial absorber can realize an absorptivity of more than 90% in a wide band of 3.53–24.00 GHz, and improve absorbing efficiency for transverse magnetic (TM) waves of oblique incidence angle from 0° to 70°. The compression test results reveal that compressive strength of the 3D printed honeycomb can reach 10.7 MPa with density of only 254.91 kg/m^3^, and the energy absorption per volume *W*_*v*_ and per unit mass *W*_*m*_ are 4.37 × 10^3^ KJ/m^3^ and 17.14 KJ/Kg, respectively. The peak compressive strength and energy absorption per mass are at least 2.2 and 3 times comparing to metallic lattice cores with the same density. Outstanding electromagnetic wave absorption and mechanical performance make the present three-dimensional metamaterial absorber more competitive in engineering applications.

## Introduction

Metamaterial absorber (MMA) is a composite metamaterial, which usually consists of periodic artificial structures and dielectric substrate. Through transforming the electromagnetic wave energy into other forms, MMA can realize electromagnetic absorption^1^. As an important branch of metamaterials, MMA has attracted great attention in the past decade, and its applications have covered many areas, such as stealth technologies, communication antennas, radars and so on^2–5^. In 2008, Landy *et al*. designed a perfect MMA through metal-dielectric composite structure, which consists of electric resonators and magnetic resonators. Their proposal realized nearly 100% absorptivity at 11.5 GHz^6^. After that, metal-dielectric composite structures are widely used in the design of MMAs in GHz and THz range^7–10^. However, this kind of MMA can only realize efficient absorption in a narrow band. In order to broaden the absorption band, multilayer structures are used^11–14^. In this way, the thickness will be so large that limits the applications of MMA. Another effective method to achieve broadband absorbing is using the frequency selective surface (FSS) absorber consisting of lossy resistive patches^15–18^. These absorbers usually have various patterns of resistive patches placing on the dielectric substrate. By reasonable design of the planar patterns, broadband absorbing performance can be easily obtained with an ultra-thin thickness. The realization of resistive FSS absorbers made a breakthrough in the research of radar absorbing.

Previous studies mostly focused on planar structure absorbers. Nowadays, researchers have extended the planar absorbers to three-dimensional structures^19–21^. In 2016, Shen *et al*. found that the folded resistive patches standing up on a metallic backboard can exhibit not only a wide-band absorbing but also wide-angle absorbing characteristic^19^. Their proposal gives a new idea for the design of MMA, especially for the large incidence angle wave absorbing. As aerospace materials, in addition to outstanding electromagnetic properties, they also need to be strong enough to resist aerodynamic force and thin enough to install on the surface of the spacecraft. However, such three-dimensional absorber may be difficult to resist deformation because of the weak mechanical properties of the thin resistive patches. Sandwich structures with honeycomb cores have excellent mechanical performances and are widely applied as engineering structures^22,23^. Recent research shows that honeycombs and its composite structures have significant benefits on energy absorption^24,25^, vibration control^26^, thermal buckling resistance^27^, acoustic absorption^28^ and even electromagnetic absorption^29^ properties. Based on these benefits, honeycomb structures can be used as multifunctional design, such as the combination of electromagnetic wave absorption, energy absorption and load carrying properties. Compared to traditional metamaterial absorbers of poor mechanical performances, the honeycomb based design may have more advantages.

**Figure 1:**
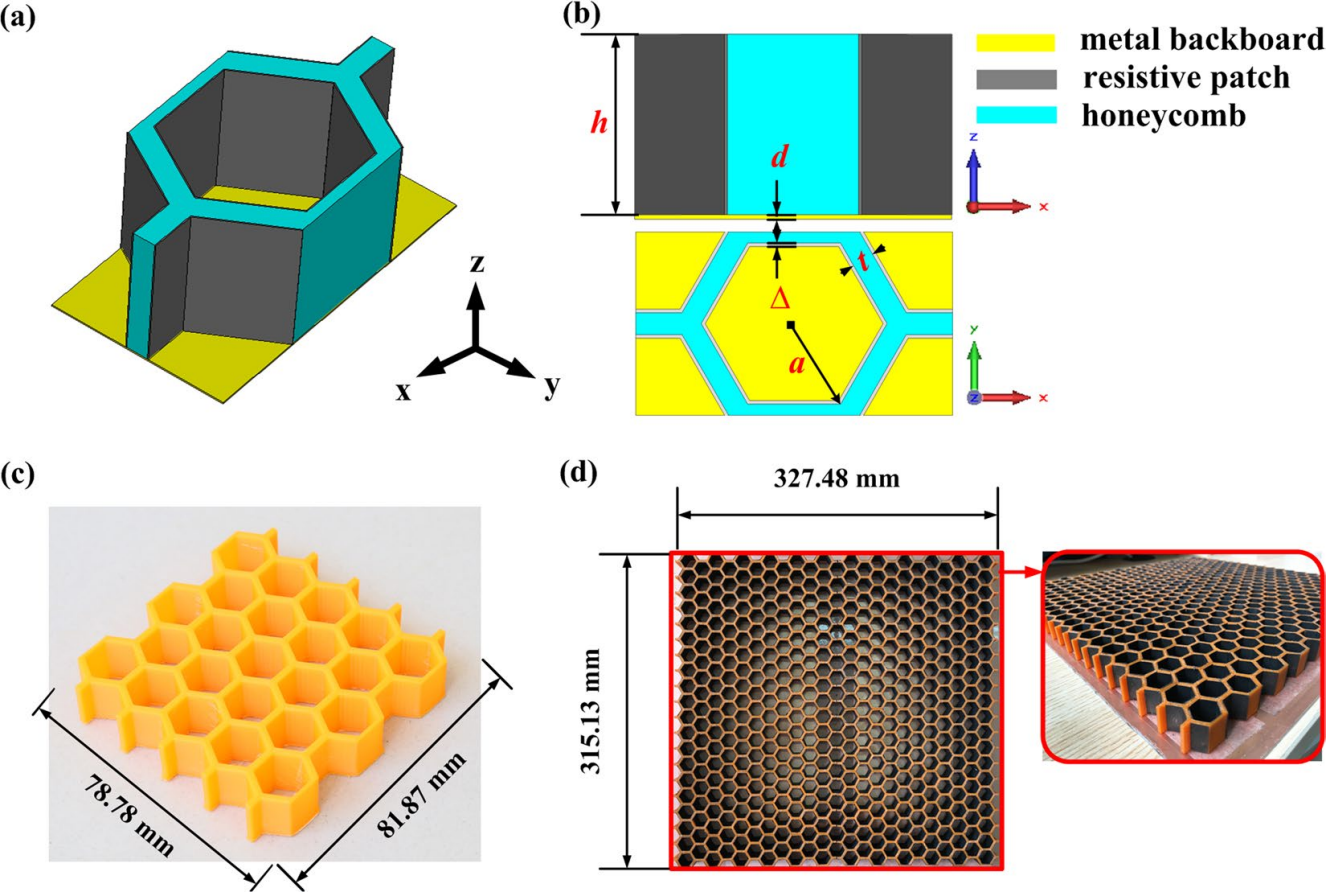
Structure design of the three-dimensional MMA. (**a**) Unit cell diagram. (**b**) View of unit cell in plane *x*-*z* and *x*-*y*. (**c**) Honeycomb sample for compressive test and (**d**) sample of the three-dimensional MMA.

In this paper, a three-dimensional MMA was proposed, which consists of hexagonal honeycombs with resistive patches attached on its walls, as shown in Fig. 1(a,b), and they are placed on a metal backboard. Figure 1(b) shows the front view and top view of its unit cell in plane *x*-*z* and *x*-*y*. The height, the maximum inner radius and the thickness of honeycomb are *h*, *a* and *t*, respectively. The material of honeycombs is polylactic acid (PLA), which is a biodegradable and bioactive thermoplastic aliphatic polyester. The dielectric constant *ε* and dielectric loss *δ* of PLA are *ε* =  3  and δ = 0.01at room temperature^30^. A copper panel is used as the backboard with thickness *d* = 1 mm. And the thickness and resistance of resistive patches are defined as Δ (Δ = 0.01 mm) and *R*_*s*_, respectively. Genetic algorithm in software, CST Microwave Studio 2015, is applied into the structure optimization. Structure parameters *h*, *a*, *t* and resistance value *R*_s_ are set as variables. Reflectivity S_11_ is set as target value. Then the optimized structure parameters can be obtained, which are *h* = 15.51 mm, *a* = 8 mm, *t* = 1.9 mm, *R*_*s*_ = 219.28 Ω/sq. Figure 1(c) shows honeycomb specimen for compressive test with a dimension of 78.78 mm × 81.78 mm. The three-dimensional MMA for electromagnetic wave absorption measurement with a global size of 315.13 mm × 327.48 mm is shown in Fig. 1(d).

## Results

### Wave absorbing properties

**Figure 2:**
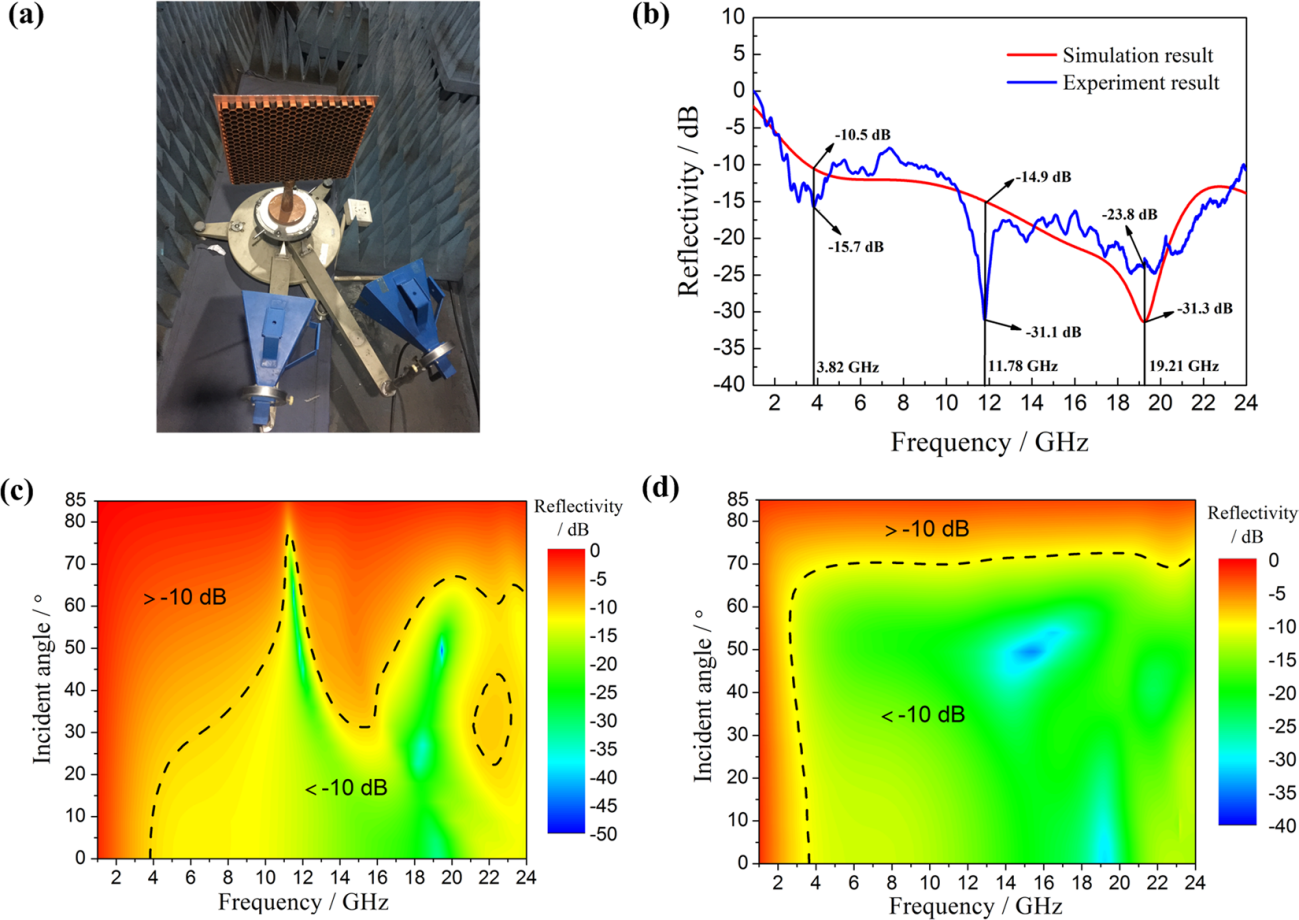
The wave absorbing property of the three-dimensional MMA. (**a**) Experiment setup. (**b**) Simulation and experiment results of reflectivity for vertical incident waves. (**c**) Reflectivity of oblique incident TE waves and (**d**) reflectivity of oblique incident TM waves.

Microwave-absorbing characteristics of the proposed MMA are shown in the following figure. The red curve in Fig. 2(b) shows the simulated reflectivity of vertical incident waves in 1–24 GHz, indicating that this three-dimensional MMA can realize an absorptivity of more than 90% in 3.53–24.00 GHz. In order to verify this simulated result, experiment was performed in microwave anechoic chamber, as shown in Fig. 2(a). Then experimental reflectivity can be obtained, as the blue curve shown in Fig. 2(b). For a metamaterial absorber, the absorptivity can be expressed as following:

1 $${\rm{A}}=1-{|{{\rm{S}}}_{{\rm{11}}}|}^{2}-{|{{\rm{S}}}_{{\rm{21}}}|}^{2}$$

In equation (1), *A* represents absorptivity, ${|{{\rm{S}}}_{{\rm{11}}}|}^{2}$ and ${|{{\rm{S}}}_{{\rm{21}}}|}^{2}$ represent reflectivity and transmissivity, respectively. In this work, because of the existence of metal backboard, ${|{{\rm{S}}}_{{\rm{21}}}|}^{2}$ equals to 0. According to equation (1), when S_11_ (in dB) is less than −10, the absorptivity will be more than 90%. Therefore, if the curves are under −10 dB, the errors of absorptivity between simulation and experimental results are less than 10%. In Fig. 2(b), three frequencies of peaks with bigger discrepancies between simulation and experimental results are selected to study the errors. From data in Table 1, it can be seen that the errors of absorptivity between simulation and experimental results are 6.8% at 3.82 GHz, 3.2% at 11.78 GHz and 0.2% at 19.2%. These errors are all in a reasonable range, and the causes of them mostly are sample processing error or experimental error.

**Table 1:** The error of absorptivity between simulation and experimental results.

Frequency/GHz	S_11_/dB		Absorptivity		
	Simulation	Experiment	Simulation	Experiment	Error
3.82	−10.5	−15.7	0.911	0.973	6.8%
11.78	−14.9	−31.1	0.968	0.999	3.2%
19.21	−31.3	−23.8	0.999	0.996	0.2%

Figure 2(c,d) show the reflectivity of transverse electric (TE) waves and TM waves in different incident angles. For TE waves, the MMA can keep a broadband absorbing feature when incident angle is less than 30°. As the increase of incident angle, absorbing effect of low frequency waves weakens and the absorbing bandwidth becomes narrow. While for TM waves, it can maintain an absorptivity of more than 90% in 3.53–24.00 GHz till 70°. The results indicate that this proposed MMA has an excellent large angle absorption characteristic for TM waves.

### Effects of material parameters

**Figure 3:**
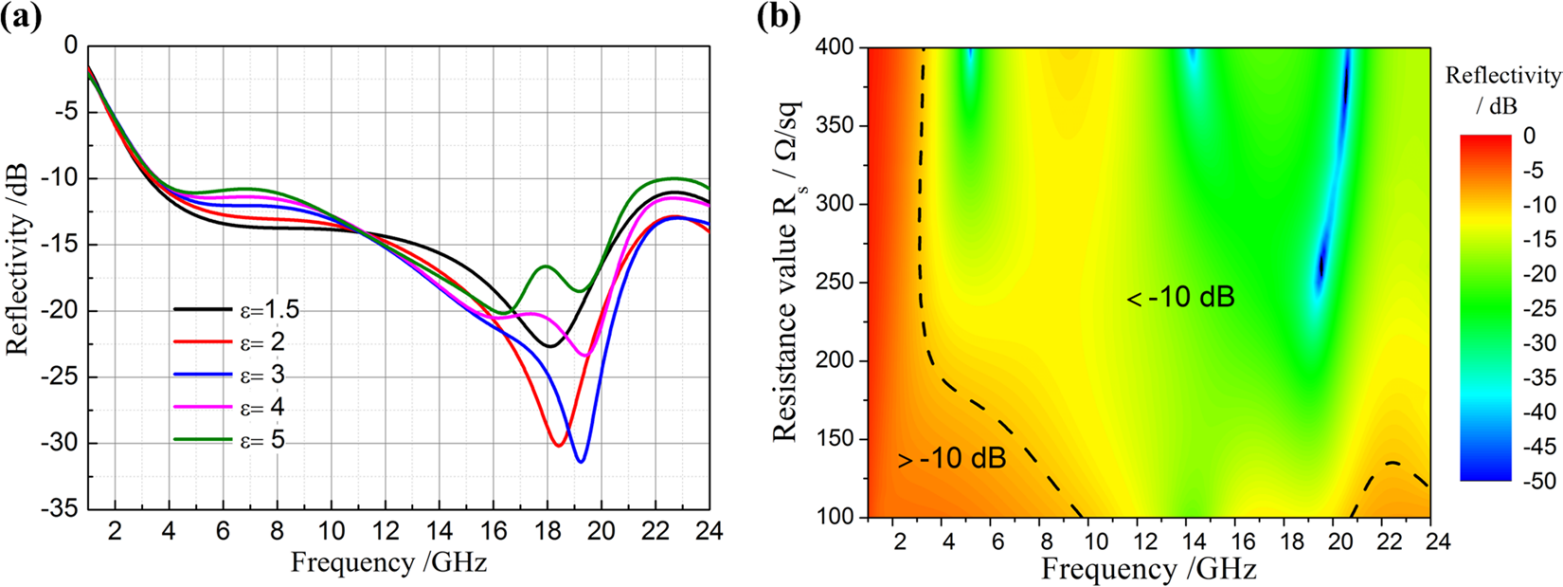
The influence of material parameters on the wave absorption performance. (**a**) The permittivity *ε* of PLA and (**b**) the resistance value *R*_*s*_ of resistive patches.

Reference^30^ has studied dielectric properties of the “as printed” PLA, indicating that the dielectric constant *ε* of PLA are depending on temperature and frequency^30^. When temperature 150 K < T < 350 K, the dielectric constant *ε* changes from 1.5–5 in 1–3 GHz. And with the increase of frequency, dielectric constant has less dependence to temperature. According to ref.^30^, five different permittivity values from 1.5 to 5 were selected to furtherly investigate the influence of dielectric constant on wave absorption performance, as shown in Fig. 3(a). The results show that as permittivity *ε* less than 5, the bandwidth of more than 90% absorptivity remains unchanged in 3.53–24.00 GHz. Due to the dielectric constant is very small, its influence on wave absorption performance can be ignored.

Another important parameter that influences absorbing property is the resistance value *R*_*s*_ of resistive patches. When a vertically incident electromagnetic wave propagates into a resistive patch with the thickness of Δ, the resistance value *R*_*s*_ of resistive patches can be expressed as following^31,32^:

2 $${{R}}_{s}=\frac{1}{{\rm{\Delta }}{\sigma }_{s}}$$

In equation (2), parameter ${\sigma }_{s}$ represents conductivity of resistive patches, which is determined by the resistance value *R*_*s*_. The resistive patches are processed using carbon paste through silk-screen printing technology and the resistance value mainly depends on the concentration of carbon paste. However, it is difficult to control the parameter *R*_*s*_ in a specific value in actual operation process. The relation between reflectivity and resistance value was studied and the results are shown in Fig. 3(b). It can be seen that as the value of *R*_*s*_ in 200–400 Ω/sq, the bandwidth of more than 90% absorptivity remains almost unchanged in 3.53–24.00 GHz. Hence, the resistive patches with resistance value in 200–400 Ω/sq can be seen as qualified samples. The preparation of resistive patches and the control of its resistance value are key steps in this research.

### Mechanical properties

**Figure 4:**
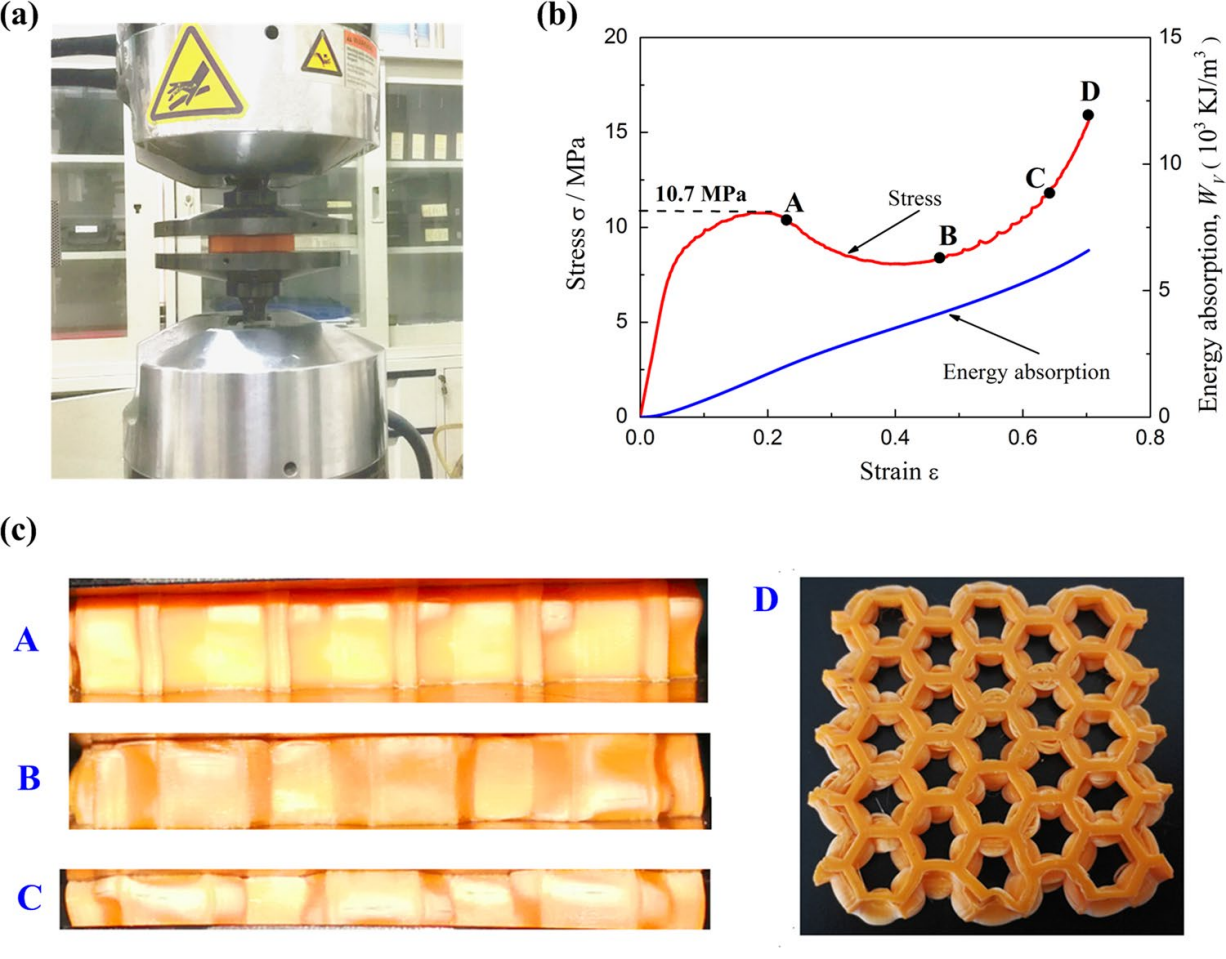
The compressive behaviors of 3D printed PLA honeycomb. (**a**) Experiment setup. (**b**) Stress and energy absorption versus compressive strain curves and (**c**) photographs illustrating the deformation history and evolution of failure at selected points marked in (**b**).

The out of plane compressive load was applied on PLA honeycomb to study mechanical properties of the proposed three-dimensional MMA, as shown in Fig. 4(a). At least 70% deformation in strain was achieved in order to record the complete deformation process. The typical measured uniaxial compressive stress versus strain response of 3D printed PLA honeycomb was shown in Fig. 4(b). The deformed images of the specimen at selected points were also shown in Fig. 4(c), and Fig. 4(c)-D shows its top view after compression. The compressive stress-strain curve is very similar to that of the aluminum foam-filled corrugated sandwich panel reported by Yan *et al*.^33^. After the initial linear elastic response with elasticity modulus of 168.55 MPa at low strains and nonlinear increase, the compressive stress reached its peak of 10.7 MPa at strain of 0.2, see Fig. 4(b). As the strain increased furthermore, the stress only slightly declined due to the soft of the 3D printed honeycomb core web, the initial formed bulges can be seen clearly in Fig. 4(c)-A. However, compared with metallic lattice cores which have a dramatic stress drop immediately after its peak, such as corrugated cores^33^, the present honeycomb has much more stabilized cores. When the strain reached about 0.42, the previous bulges propagated and contacted with the newly formed ones which require much higher compressive load, and causing the increase of stress with further increase in strain till densification. The bulges propagation and generation are showing in Fig. 4(c), A–C. Top view of specimen after compression indicates that every cell wall of the honeycomb have bulge generation and propagation, Fig. 4(c)-D.

The energy absorption capacity may be characterized by the area under the uniaxial compressive stress versus strain curve as shown in Fig. 4(b). The energy absorbed per unit volume, *W*_*v*_, was defined as:

3 $${W}_{v}={\int }_{0}^{\bar{\varepsilon }}{\sigma }{\rm{d}}{\varepsilon }$$

In addition, as mass was critical for energy absorbers for weight sensitive applications, the specific absorbed energy (SAE) (or absorbed energy per unit mass) was another important parameter. The absorbed energy per unit mass, *W*_*m*_, may be defined as:

4 $${W}_{m}={W}_{v}/{\rho }_{c}$$

where ${\rho }_{c}={\rm{254.91}}$ kg/m^3^ was the average density of the 3D printed PLA honeycomb. When $\bar{\varepsilon }={\rm{0.5}}$ was adopted, the *W*_*v*_ and *W*_*m*_ were calculated as 4.37 × 10^3^ KJ/m^3^ and 17.14 KJ/Kg, respectively.

### Comparison with competing core designs

**Figure 5:**
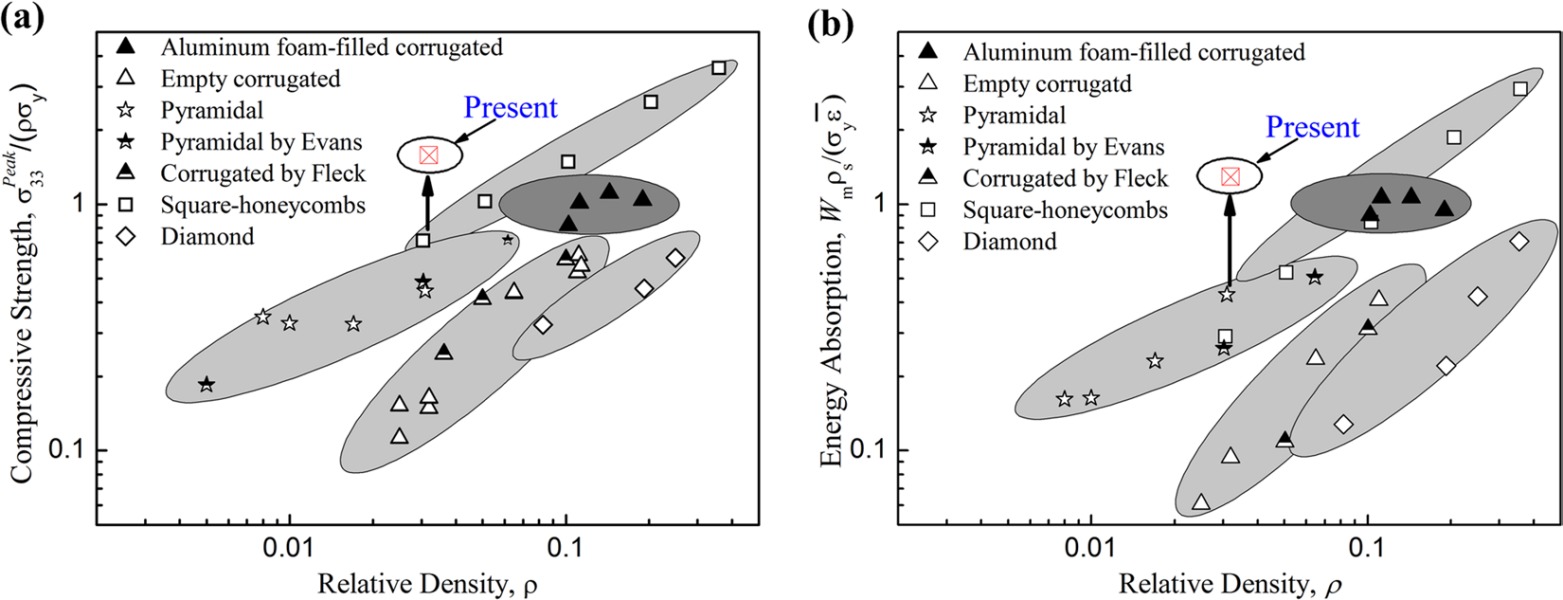
Comparison of the present 3D printed honeycomb and competing sandwich core designs^34^. (**a**) Peak compressive strength and (**b**) specific energy absorption. The arrow lines show the advantages of present results compared with metallic lattice cores with similar relative densities.

Yan *et al*. reported a novel metallic sandwich panel with aluminum foam-filled corrugated cores, and it’s remarkable advantages in compressive strength and energy absorption as well as bending resistance properties have been demonstrated^33,34^. The non-dimensional peak compressive strength and specific absorbed energy (SAE) of aluminum foam-filled corrugated core was found to be more competitive compared with the typical metallic lattice cores, such as empty corrugated, diamond, square-honeycomb and pyramidal truss cores^33^. The experimental data of the present 3D printed honeycomb were added and compared in Fig. 5(a,b). Both peak strength and SAE performances of the present honeycomb have significant advantages compared with aluminum foam-filled corrugated core and other competing lattice cores. Figure 5 reveals that the peak strength of 3D printed honeycomb is 2.2 times of metallic square honeycombs while the SAE value is 3 times of that of pyramidal cores with the similar relative density, as the arrow lines shown in Fig. 5, however, compared with other metallic lattice cores, it is even much more competitive. Furthermore, compared with previous three-dimensional absorbers^19–21^, the 3D printed honeycomb MMA have more advantages due to its excellent mechanical performances.

## Conclusion

A three-dimensional MMA consisting of PLA based 3D printed honeycomb, resistive patches and metallic backboard is reported in this paper. The experiment and simulation results indicate that the proposed MMA can realize an absorptivity of more than 90% in 3.53–24.00 GHz for vertical incident waves, and can keep this excellent absorbing effect for oblique incident TM waves from 0°–70°. Out of plane compressive load was applied on the PLA honeycomb core to study its mechanical performances. Experiment results show that the peak strength and specific energy absorption of the MMA is much more competitive which are no less than 2.2 and 3 times respectively compared with the competing metallic lattice core designs under equivalent densities. The outstanding electromagnetic wave absorption (wide band and large angle) and excellent mechanical performances (high specific strength and energy absorption) of the multifunctional designed MMA make it have great potential application as novel lightweight structural materials and electromagnetic wave absorbers.

## Methods

### Simulation

Electromagnetic simulations were performed using a commercially available software package, CST Microwave Studio 2015. The S parameters were simulated using the Frequency Domain Solver with periodic boundary conditions along the *x*, *y* and *z* directions. In the simulation, periodic boundary conditions in the *x* and *y* directions were used with open boundary conditions in the *z* direction.

### Fabrication

The PLA honeycombs were fabricated through 3D printing technology on the MakerBot Replicator platform. The specimens for compressive test have a dimension of 78.78 mm × 81.78 mm. The resistive patches were printed on a plastic film through silk-screen printing technology and then cut into small pieces. They were pasted on the inner wall of honeycomb and combined with a copper backboard. Then the three-dimensional MMA for electromagnetic wave absorption measurement with a global size of 315.13 mm × 327.48 mm can be obtained.

### Experiment

The experimental study of absorption was performed by arch measurement system in a microwave anechoic chamber. The system is based on an Agilent 8720ET network analyzer with a pair of broadband horn antennas working in the measurement frequency range. Two antennas were used for transmitting and receiving signals, respectively. Then the measured S parameters can be obtained.

The compressive tests were carried out through a hydraulic testing machine (MTS) at ambient temperature. The loading rate in the compression tests was fixed at 0.5 mm/min with a nominal strain rate less than 10^−3^ s^−1^ to ensure quasi-static compressive load was carried. The deformation history of the PLA honeycomb was acquired by a video camera.
